# A Score for Risk of Thrombolysis-Associated Hemorrhage Including Pretreatment with Statins

**DOI:** 10.3389/fneur.2018.00074

**Published:** 2018-02-16

**Authors:** Hebun Erdur, Alexandros Polymeris, Ulrike Grittner, Jan F. Scheitz, Serdar Tütüncü, David J. Seiffge, Heinrich J. Audebert, Christian H. Nolte, Stefan T. Engelter, Andrea Rocco

**Affiliations:** ^1^Department of Neurology, Charité—Universitätsmedizin Berlin, Berlin, Germany; ^2^Berlin Institute of Health, Berlin, Germany; ^3^Department of Neurology and Stroke Center, University of Basel, University Hospital Basel, Basel, Switzerland; ^4^Center for Stroke Research, Charité—Universitätsmedizin Berlin, Berlin, Germany; ^5^Department for Biostatistics and Clinical Epidemiology, Charité—Universitätsmedizin Berlin, Berlin, Germany; ^6^NeuroCure, Cluster of Excellence, Charité—Universitätsmedizin Berlin, Berlin, Germany; ^7^Neurorehabilitation Unit, University of Basel, University Center for Medicine of Aging and Rehabilitation, Felix Platter Hospital, Basel, Switzerland

**Keywords:** ischemic stroke, thrombolysis, hemorrhage, stroke outcome, predictive models

## Abstract

**Background:**

Symptomatic intracranial hemorrhage (sICH) after intravenous thrombolysis with recombinant tissue-plasminogen activator (rt-PA) for acute ischemic stroke is associated with a poor functional outcome. We aimed to develop a score assessing risk of sICH including novel putative predictors—namely, pretreatment with statins and severe renal impairment.

**Methods:**

We analyzed our local cohort (Berlin) of patients receiving rt-PA for acute ischemic stroke between 2006 and 2016. Outcome was sICH according to ECASS-III criteria. A multiple regression model identified variables associated with sICH and receiver operating characteristics were calculated for the best discriminatory model for sICH. The model was validated in an independent thrombolysis cohort (Basel).

**Results:**

sICH occurred in 53 (4.0%) of 1,336 patients in the derivation cohort. Age, baseline National Institutes of Health Stroke Scale, systolic blood pressure on admission, blood glucose on admission, and prior medication with medium- or high-dose statins were associated with sICH and included into the risk of intracranial hemorrhage score. The validation cohort included 983 patients of whom 33 (3.4%) had a sICH. c-Statistics for sICH was 0.72 (95% CI 0.66–0.79) in the derivation cohort and 0.69 (95% CI 0.60–0.77) in the independent validation cohort. Inclusion of severe renal impairment did not improve the score.

**Conclusion:**

We developed a simple score with fair discriminating capability to predict rt-PA-related sICH by adding prior statin use to known prognostic factors of sICH. This score may help clinicians to identify patients with higher risk of sICH requiring intensive monitoring.

## Introduction

Symptomatic intracranial hemorrhage (sICH) occurs in 2–4% of patients receiving intravenous recombinant tissue-plasminogen activator (rt-PA) for acute ischemic stroke (1–3). sICH is associated with poor clinical outcome and a high fatality rate of approximately 50% (4).

Therefore, the accurate identification of patients with higher risk of rt-PA-related hemorrhage is of high relevance. Several predictive instruments for estimated risk of sICH after thrombolytic therapy have been developed in recent years (e.g., HAT, MSS, THRIVE, SITS-sICH, SEDAN, and TURN) (5–10). However, they showed modest to moderate discriminatory ability only and it has been suggested that the accuracy of scores may be improved by inclusion of additional risk factors (11). Indeed, novel putative risk factors for sICH have been identified, namely, severe renal impairment and prior use of medium to high-dose statins (12, 13), which are not part of existing sICH scores. Furthermore, some of the existing scores rely on identification of early ischemic changes on computer tomography (CT), requiring trained stroke neurologists or neuroradiologists (14). Moreover, scores including early ischemic changes on CT are not applicable in MRI-based thrombolysis. We therefore aimed to develop a simple score based on history, clinical, and laboratory data, which may be used in clinical settings without quick and reliable assessment of early signs of infarction on CT. Specifically, we aimed to evaluate whether inclusion of severe renal impairment and prior use of statins can improve the discrimination of sICH in a predictive score.

## Materials and Methods

### Study Population and Data Acquisition

From January 2006 to March 2016, all consecutive patients with acute ischemic stroke admitted to our tertiary care center (Department of Neurology, Charité—Universitätsmedizin Berlin, Campus Benjamin Franklin) and undergoing intravenous thrombolysis were included in a prospective database. Our prospective database was approved by the ethics committee of the Charité—Universitätsmedizin Berlin (Institutional Review Board number EA4/061/14). An ethics committee approval was not required for the current analysis, in accordance with laws and regulations in the Federal State of Berlin. Stroke severity on admission was assessed using the National Institutes of Health Stroke Scale (NIHSS) by a trained neurologist. Demographics, vascular risk factors (applying criteria used in prior research), admission blood pressure, glucose levels, creatinine, estimated glomerular filtration rate (eGFR), time from onset of stroke symptoms until treatment, previous medication including statin, and anti-platelet use were recorded. Simvastatin 40 mg and simvastatin 80 mg (or equivalents, see Table S1 in Supplementary Material) were defined as medium- and high-dose statin use, respectively (12, 15, 16). Renal impairment was assessed by eGFR using the CKD-EPI formula (13). Patients underwent neuroimaging before treatment and routinely 24–36 h after thrombolysis or earlier in case of deterioration. Brain images were analyzed by trained neuroradiologists. Presence of symptomatic hemorrhage according to criteria of the European-Australasian Acute Stroke Study (ECASS-III) was recorded (17). The main outcome parameter was the occurrence of sICH after thrombolysis.

### Data Analysis, Score Derivation, and Score Validation

For categorical variables, proportions were calculated by dividing the number of events by the total number of patients, excluding missing or unknown cases. For continuous variables, median and interquartile ranges (IQR) were calculated.

We developed a score based on previous methodological recommendations (18). We performed a PubMed search to identify associated factors with sICH in patients treated with rt-PA. Based on reviews (19) and other published predictive scores for sICH (5–10), following factors were identified: older age, history of hypertension, ischemic heart disease, atrial fibrillation, congestive heart failure, diabetes, renal impairment, treatment with antiplatelets prior to stroke, treatment with statins prior to stroke, severity of stroke measured by NIHSS, higher serum glucose levels, higher systolic blood pressure on admission, early signs of ischemic infarction on CT, leukoaraiosis, higher levels of triglycerides, higher weight, aPTT, prestroke mRS score, and longer time to treatment. Early CT signs of ischemic stroke, leukoaraiosis, triglycerides, aPTT, and prestroke mRS score were not registered in our database and therefore not considered in score derivation. The χ^2^ test was used for comparison of categorical variables. Comparisons of continuous variables were performed using the Mann–Whitney *U*-test. All factors with a *P*-value ≤0.10 were included in a multiple backward stepwise logistic regression analysis in order to identify characteristics that where associated with sICH. Based on the β-coefficients of the final multiple binary logistic regression model, we calculated the area under ROC (c-statistics) and 95% CI of the score as a measure of discrimination of sICH. The resulting risk of sICH score (RICH score) was validated in an independent cohort of ischemic stroke patients treated with intravenous thrombolysis from the University Hospital Basel, Switzerland. Comparison with the previously published scores MSS (6) and THRIVE (7) were performed in the independent validation cohort. To facilitate the use of the score in the clinical setting, we additionally developed a final ordinal score based on the results of the multiple logistic regression analysis. For this, continuous variables were dichotomized and the optimal cutoff points were determined by choosing those cutoff points which yielded the highest odds ratios. Every item on the RICH score was assigned with 0 or 1 point. All tests were two-tailed and statistical significance was determined at an alpha level of 0.05. All statistical analyses were performed using SPSS (Version 22.0) and STATA (Version 14.2).

## Results

### Baseline Characteristics

From 2006 to 2016, a total of 1,742 acute stroke patients were treated at the Charité (Berlin) stroke unit with rt-PA and included in our prospective database. Patients with a diagnosis of a stroke mimic at discharge (72; 4.1%), additional intraarterial thrombolysis and/or thrombectomy (228; 13.1%), and patients with unknown stroke onset and onset-to-treatment time >270 min (130; 7.5%) were not included in our analysis. A total of 1,336 patients were eligible for analysis and score development (24 patients with stroke of unknown onset underwent additional thrombectomy). Table 1 lists baseline characteristics of patients with and without sICH.

**Table 1:** Demographics and baseline characteristics with univariate associations of patients with and without symptomatic intracranial hemorrhage (sICH) in the derivation cohort (Berlin).

	No sICH*n* = 1,283	sICH*n* = 53	*P*-value
Age, median [interquartile ranges (IQR)]	75 (67–83)	80 (72–87)	0.003
Sex, female (%)	623 (48.6)	27 (50.9)	0.73
Arterial hypertension (%)	1,066 (83.6)	46 (90.2)	0.21
Atrial fibrillation (%)	475 (37.4)	23 (45.1)	0.26
Diabetes mellitus (%)	309 (24.3)	18 (35.3)	0.07
Hyperlipidemia (%)	676 (53.6)	28 (56.0)	0.74
Previous stroke or transient ischemic attack (%)	339 (26.7)	14 (27.5)	0.90
Coronary artery disease (%)	260 (20.4)	13 (25.5)	0.38
Antiplatelets (%)	523 (42.7)	29 (58.0)	0.03
Statins (%)^a^	165 (12.9)	13 (24.5)	0.01
National Institutes of Health Stroke Scale (NIHSS), median (IQR)	8 (4–15)	10 (7–19)	0.008
NIHSS ≥5 (%)	830 (64.7)	44 (83.0)	0.06
Onset-to-treatment time (min), median (IQR)	112 (85–151)	126 (92–155)	0.31
Systolic blood pressure (mmHg), median (IQR)	152 (136–172)	160 (148–181)	0.04
Glucose (mg/dL), median (IQR)	121 (107–148)	138 (119–171)	0.001
Estimated glomerular filtration rate (eGFR), median (IQR)	66 (49–81)	62 (47–81)	0.29
Severe renal impairment (eGFR ≤ 30) (%)	64 (5.0)	5 (9.4)	0.15

*^a^No or low-dose statins vs. medium or high-dose statins*.

### Risk Factors of Intracranial Hemorrhage and Score Development

Symptomatic ICH according to ECASS-III-criteria occurred in 53 patients (4.0%). In multiple logistic regression analysis, older age, higher stroke severity measured by NIHSS upon admission, higher blood glucose upon admission, and treatment with medium or high-dose statins before the acute event were associated with sICH (Table 2). Systolic blood pressure upon admission was added into the model according to the literature. Table 2 lists all items of the resulting RICH score. The c-statistics of the RICH score for discrimination of sICH was 0.72 (95% CI 0.66–0.79) in the derivation cohort.

**Table 2:** Multiple regression analysis with β-coefficients for risk factors associated with symptomatic intracranial hemorrhage in the final model for the derivation group (*n* = 1,244), AUC: 0.72 (95% CI: 0.65–0.79).

Item	β-coefficient (95% CI)	*P*-value
Age	0.034 (0.006–0.06)	0.02
National Institutes of Health Stroke Scale on admission	0.041 (0.001–0.08)	0.04
Glucose on admission	0.008 (0.003–0.01)	0.001
Systolic blood pressure on admission	0.01 (−0.0003 to 0.02)	0.057
Pretreatment with medium or high-dose statins	0.987 (0.32–1.66)	0.004
Constant term	−9.134 (−11.96 to −6.31)	<0.001

### Validation and Comparison of Scores

The score was then validated and compared with other scores in the thrombolysis registry of the University Hospital Basel, which included 983 patients of whom 33 (3.4%) had a sICH. Table 3 shows the c-statistics for the RICH score and other scores in the validation cohort. The RICH score discriminated sICH in the validation cohort with a c-statistic of 0.69 (95% CI 0.60–0.77). There was no significant difference regarding discriminatory ability compared to the MSS and THRIVE scores. We also assessed the ability to discriminate sICH using alternative models with different combinations of additional variables including severe renal impairment. None resulted in significant changes of the discriminative ability of the score.

**Table 3:** c-Statistics of the RICH, MSS, and THRIVE scores for risk of symptomatic intracranial hemorrhage (sICH) after systemic thrombolytic therapy for acute ischemic stroke in the validation cohort (Basel, *n* = 983).

Score	sICH per ECASS-III^a^
RICH (*n* = 983)	0.69 (0.60–0.77)
MSS (*n* = 969)	0.66 (0.52–0.70)
THRIVE (*n* = 979)	0.67 (0.59–0.76)

*^a^c-Statistics (95% CI)*.

**Figure 1:**
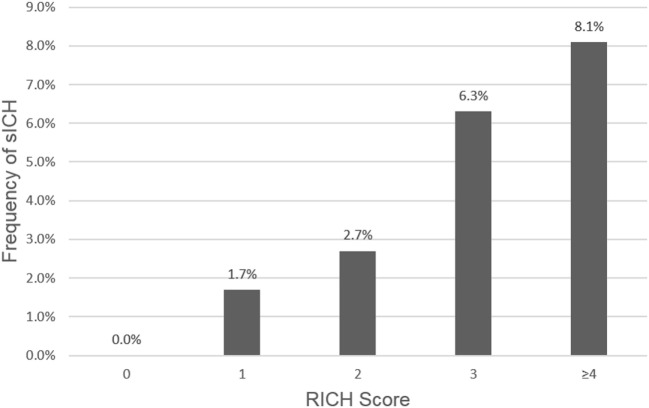
Frequency of symptomatic intracranial hemorrhage (sICH) according to points on the RICH score. Patients at risk: 0 points = 6.7%, 1 point = 25.0%, 2 points = 33.3%, 3 points = 25.9%, ≥4 points = 9.0%.

For ease of use of the RICH score, we developed a risk calculator giving the probability of sICH after thrombolysis based on individual patient data (see Data Sheet S1 in Supplementary Material). In addition, we developed a final ordinal score based on the results of the multiple logistic regression analysis to further facilitate the use of the score in the clinical setting. For this, we dichotomized continuous variables and assigned one point for each risk factor (Table 4). Figure 1 shows the frequency of sICH according to points on the RICH score.

**Table 4:** Multiple logistic regression analysis with odds ratios for dichotomized risk factors associated with symptomatic intracranial hemorrhage in the final model and assigned points for each variable.

Item	OR (95% CI)	*P*-value	Points
Age >80 years	1.8 (1.03–3.3)	0.04	1
National Institutes of Health Stroke Scale ≥5	2.4 (1.1–5.0)	0.02	1
Glucose >125 mg/dL	2.5 (1.4–4.5)	0.003	1
Systolic blood pressure >155 mmHg	2.0 (1.1–3.3)	0.02	1
Pretreatment with medium or high-dose statins^a^	2.6 (1.3–5.1)	0.005	1

*^a^Medium or high-dose statins: atorvastatin (10, 20, 40, and 80 mg), rosuvastatin (5, 10, and 20 mg), simvastatin (40 and 80 mg) (see Table S1 in Supplementary Material for a complete list of statins)*.

## Discussion

The RICH score is an easy and quick computable score for the risk of sICH in patients with an acute stroke treated with intravenous rt-PA. Its simplicity and fair discriminatory ability may help clinicians to identify patients with higher or low risk of sICH after rt-PA. All items of the score are easy and quick to obtain and do not require interpretation. While incorporating well-known risk factors of sICH such as age, stroke severity, and admission levels of blood glucose, our score differs from other published scores in considering pretreatment with medium or high-dose statins as additional risk factor. Furthermore, in contrast to many other scores, the RICH score does not include early signs of ischemic infarction on CT, which may facilitate its use.

Several scores for the risk of sICH after rt-PA have been published in recent years (5–10). Most involve established risk factors of sICH such as age (MSS, SEDAN, SITS-sICH, and THRIVE), stroke severity (MSS, HAT, SEDAN, SITS-sICH, and THRIVE), level of blood glucose on admission (MSS, HAT, SEDAN, and SITS-sICH), and early signs of ischemic infarction on CT (HAT and SEDAN). The accuracy of these previously published scores is therefore overall comparable. In studies comparing scores for sICH, the c-statistic ranged mostly between 0.60 and 0.80, suggesting the accuracy was modest to good (20, 21). Therefore, identifying additional risk factors of sICH may improve the accuracy of predictive scores, making them more precise (22, 23). Our study shows that preexisting treatment with medium and high-dose statins is possibly an important additional risk factor and should be considered in future studies investigating rt-PA-related hemorrhage. Inclusion of severe renal impairment on the other hand did not improve the discriminatory ability of the score. This may be attributable to a small effect size of renal impairment on sICH, which may have precluded detection of an effect in the current cohorts due to the relatively low number of patients with sICH.

Risk factors for sICH after thrombolysis such as stroke severity, onset-to-treatment time, and early signs of infarction on CT may reflect the extent and progression of ischemic injury to the blood–brain barrier. Other risk factors such as stroke-associated hyperglycemia and hypertension may represent an acute stroke-related stress reaction (24, 25), and there is debate whether stroke-associated hyperglycemia is merely an epiphenomenon (and a marker) or a cause of poor outcome and sICH (22, 26). In contrast to these risk factors, the novel candidate risk factor pretreatment with statins may contribute to a higher risk of clinically relevant hemorrhage after rt-PA *via* different mechanisms. More specifically, a higher dose-dependent risk of cerebral hemorrhage after thrombolytic therapy may be attributable to antithrombotic and anticoagulative properties of statins (27, 28).

It is important to note that a higher risk of sICH on a score alone does not justify withholding treatment with rt-PA (29). Whiteley et al. have shown that patients with higher predicted risk of sICH still had a clinically relevant positive effect from therapy with rt-PA (11). Instead, the RICH score may be an additional tool to (a) identify patients who may benefit from intensified monitoring of putative complications and (b) to identify patients with a very low risk of sICH (provided that there are no contraindications for rt-PA). The RICH score may possibly represent an alternative to established scores in clinical settings where a fast and reliable assessment of early ischemic changes on CT is not available. All the items of the RICH score are part of the routine check-up for stroke patients that are suitable for rt-PA, and therefore easily available and quick to perform. Comparison with existing scores (namely MSS and THRIVE) proved a fair discriminatory ability of the RICH score.

Although we derived our score using data from a large single-center cohort and sICH rates were similar to previous studies (1, 2, 30), the number of patients with sICH was small. This may have led to over fitting and thus over-optimism regarding the goodness of fit of our final model in our derivation cohort. However, validation of the RICH score in an independent stroke cohort showed a significant predictive value.

## Conclusion

The RICH score may help clinicians to identify patients with low and higher risk of rt-PA related sICH. Inclusion of novel candidate risk factors for sICH such as pretreatment with medium or high-dose statins may improve the accuracy of predictive instruments. However, a higher risk of sICH on the RICH score alone should not preclude patients from thrombolysis.

## Author Contributions

HE and AR had full access to all of the data in the study and take responsibility for the integrity of the data and the accuracy of the data analysis. Study concept and design: HE and AR. Acquisition of data: HE, AP, JS, ST, and DS. Analysis and interpretation of data: HE, UG, and AR. Drafting of the manuscript: HE. Critical revision for important intellectual content: HE, AP, UG, JS, ST, DS, HA, CN, SE, and AR. Study supervision: HE and AR.

## Conflict of Interest Statement

HE, AP, JS, UG, and ST report no disclosures. DS has received funding from the Swiss Heart Foundation, the Science Funds of the University Hospital Basel, and the University of Basel and has served on scientific advisory boards for Bayer and Pfizer. HA reports receiving speaker honoraria from Boehringer-Ingelheim, and speaker and consultancy honoraria from Lundbeck A/S. CN has received funding for travel or speaker honoraria from Bayer, BI, Gore, Sanofi, and BMS/Pfizer. SE has received funding for travel or speaker honoraria from Bayer and Boehringer-Ingelheim, he has served on scientific advisory boards for Bayer, Boehringer-Ingelheim, BMS/Pfizer, and Covidien and on the editorial board of Stroke. He has received an educational grant from Pfizer and research support from the Science Funds (Wissenschaftsfonds) of the University Hospital Basel, the University Basel, the Swiss Heart Foundation, and the Swiss National Science Foundation. AR has received honoraria for lectures from Bayer and Ever Pharma.
