# Supplementary material for: A Score for Risk of Thrombolysis-Associated Hemorrhage Including Pretreatment with Statins
Source: Front Neurol. 2018 Feb 16;9:74. doi: 10.3389/fneur.2018.00074 (PMC5820302; doi:10.3389/fneur.2018.00074)
Supplement: Supplementary file 2 [file Table_1.docx]

ONLINE SUPPLEMENT

Supplemental table

Table 1 – Classification of statin doses according to reduction of LDL-cholesterol

| Statin | Low dose (LDL reduction <35%) | Medium dose (LDL reduction 35-44%) | High dose (LDL reduction ≥45%) |
| --- | --- | --- | --- |
| Atorvastatin |  | 10, 20 mg | 40, 80 mg |
| Fluvastatin | 20, 40, 80 mg |  |  |
| Pravastatin | 10, 20, 40, 80 mg |  |  |
| Rosuvastatin |  | 5, 10 mg | 20 mg |
| Simvastatin | 5, 10, 20 mg | 40 mg | 80 mg |

LDL indicates low density lipoprotein. Data derived from Scheitz JF, Seiffge DJ, Tütüncü S, Gensicke H, Audebert HJ, Bonati LH, et al. Dose-Related Effects of Statins on Symptomatic Intracerebral Hemorrhage and Outcome After Thrombolysis for Ischemic Stroke. Stroke. 2014;45:509-514.
